# Severely ill COVID-19 patients display impaired exhaustion features in SARS-CoV-2-reactive CD8^+^ T cells

**DOI:** 10.1126/sciimmunol.abe4782

**Published:** 2021-01-21

**Authors:** Anthony Kusnadi, Ciro Ramírez-Suástegui, Vicente Fajardo, Serena J Chee, Benjamin J Meckiff, Hayley Simon, Emanuela Pelosi, Grégory Seumois, Ferhat Ay, Pandurangan Vijayanand, Christian H Ottensmeier

**Affiliations:** 1 La Jolla Institute for Immunology, La Jolla, CA 92037.; 2 NIHR and CRUK Southampton Experimental Cancer Medicine Center, Faculty of Medicine, University of Southampton, Southampton, UK.; 3 Southampton Specialist Virology Centre, Department of Infection, University Hospital Southampton NHS Foundation Trust, Southampton, UK.; 4 Liverpool Head and Neck Center, Institute of Translational Medicine, University of Liverpool & Clatterbridge Cancer Center NHS Foundation Trust, Liverpool, UK.; 5 Department of Medicine, University of California San Diego, La Jolla, CA 92037.

## Abstract

The molecular properties of CD8^+^ T cells that respond to SARS-CoV-2 infection are not fully known. Here, we report on the single-cell transcriptomes of >80,000 virus-reactive CD8^+^ T cells, obtained using a modified Antigen-Reactive T cell Enrichment (ARTE) assay, from 39 COVID-19 patients and 10 healthy subjects. COVID-19 patients segregated into two groups based on whether the dominant CD8^+^ T cell response to SARS-CoV-2 was ‘exhausted’ or not. SARS-CoV-2-reactive cells in the exhausted subset were increased in frequency and displayed lesser cytotoxicity and inflammatory features in COVID-19 patients with mild compared to severe illness. In contrast, SARS-CoV-2-reactive cells in the dominant non-exhausted subset from patients with severe disease showed enrichment of transcripts linked to co-stimulation, pro-survival NF-κB signaling, and anti-apoptotic pathways, suggesting the generation of robust CD8^+^ T cell memory responses in patients with severe COVID-19 illness. CD8^+^ T cells reactive to influenza and respiratory syncytial virus from healthy subjects displayed polyfunctional features and enhanced glycolysis. Cells with such features were largely absent in SARS-CoV-2-reactive cells from both COVID-19 patients and healthy controls non-exposed to SARS-CoV-2. Overall, our single-cell analysis revealed substantial diversity in the nature of CD8^+^ T cells responding to SARS-CoV-2.

## Teaser

Fewer SARS-CoV-2-reactive memory CD8^+^ T cells with ‘exhaustion’ features are present in COVID-19 patients with severe vs. mild disease.

## INTRODUCTION

Coronavirus (CoV) infections with SARS-CoV-2 have created a global crisis; a large international effort is underway to develop treatments and vaccines to reduce the severity of disease and to provide protective immunity. To inform this effort, a detailed understanding of anti-viral immune responses is required. CD8^+^ T cell responses are thought to be critical for control of viral infections (*1*–*4*), but to date our understanding of anti-viral CD8^+^ T cell responses, specifically against Coronaviridae during infection and in the memory phase, is limited. Recently, studies have begun to improve our knowledge about CD8^+^ T cells against SARS-CoV-2 (*5*–*14*), but the molecular features that associate with poor clinical outcomes or differentiate them from other virus-reactive CD8^+^ T cells remain incompletely understood. Furthermore, despite the recent progress in understanding the biology of SARS-CoV-2-reactive and cross-reactive CD8^+^ T cells (*5*, *8*, *9*, *12*, *14*–*23*), comprehensive unbiased global profiling of SARS-CoV-2-reactive and cross-reactive CD8^+^ T cells has not been performed to date. Here, we report on data generated by single-cell RNA sequencing of virus-reactive CD8^+^ T cells from COVID-19 patients with different clinical severity. We benchmark these data against the transcriptomes from CD8^+^ T cells from healthy donors, who have memory responses to other respiratory viruses.

## RESULTS

### Evaluation of virus-reactive CD8^+^ T cells

**Fig. 1:**
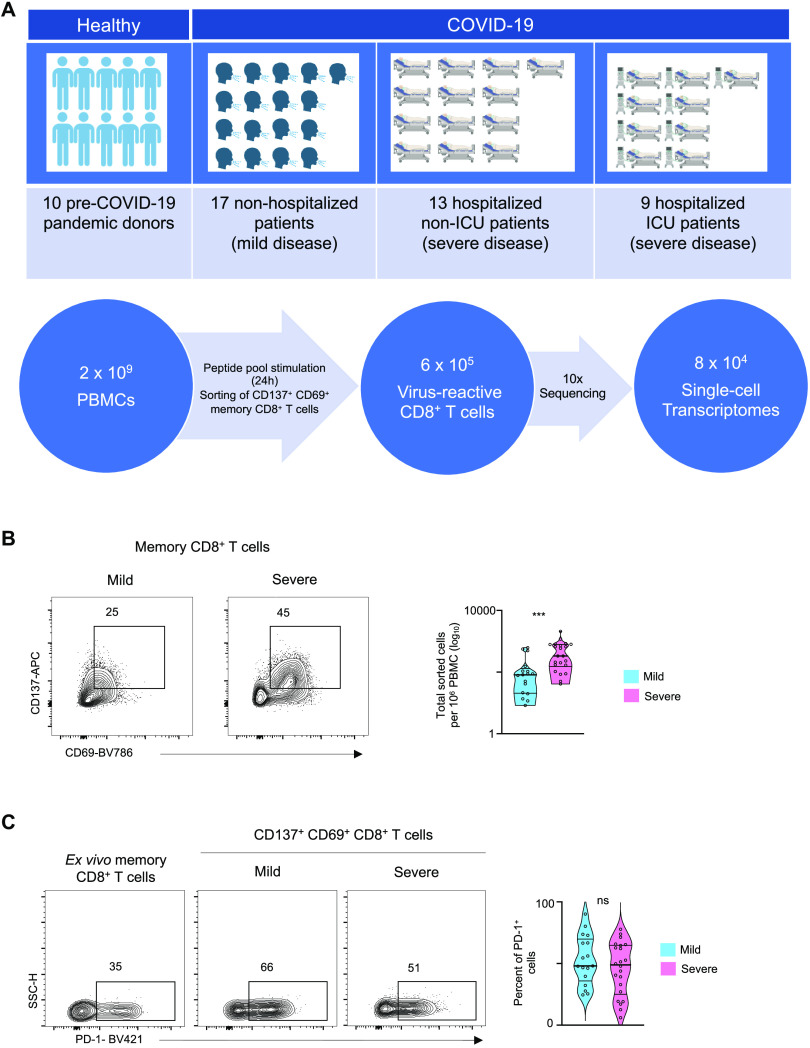
CD8^+^ T cell responses in COVID-19 illness. (**A**) Study design overview. (**B**) Representative FACS plots displaying surface staining of CD137 and CD69 in post-enriched CD8^+^ memory T cells, stimulated for 24 hours with SARS-CoV-2 peptide pools, from COVID-19 patients with mild and severe illness (left), and summary of the number of cells sorted per million PBMC (right); data are displayed as median with interquartile ranges for 17 and 22 patients with mild and severe COVID-19 disease, respectively. (**C)** Representative FACS plots (left) showing surface expression of PD-1 in CD8^+^ memory T cells ex vivo (without in vitro stimulation) and in CD137^+^CD69^+^ CD8^+^ memory T cells following stimulation, post-enrichment (CD137-based) and corresponding summary plots (right) showing proportion of PD-1 expressing cells in each study subject (*P* = 0.26, unpaired *t*-test); data are displayed as median with interquartile ranges for 17 mild and 22 severe COVID-19 patients, respectively. ***P<0.001 by Mann-Whitney test (**B**).

From 39 subjects with confirmed SARS-CoV-2 infection (17 patients with relatively milder disease not requiring hospitalization, 13 hospitalized patients and 9 additional patients requiring intensive care unit support (ICU)) (Fig. 1A and table S1), we isolated virus-reactive CD8^+^ memory T cells using a modified Antigen-Reactive T cell Enrichment (ARTE) assay (*24*–*26*). Peripheral blood mononuclear cells (PBMC) were first stimulated in vitro for 24 hours with peptide pools specific to SARS-CoV-2 (Methods and (*7*, *8*)) and responding CD8^+^ memory T cells, hereafter referred to as viral-reactive cells, were then isolated based on the expression of the cell surface activation markers CD137 and CD69 (Fig. 1A,B and fig. S1A) (*5*, *8*, *9*). We observed that the numbers of SARS-CoV-2-reactive memory CD8^+^ T cells were significantly increased in patients with severe COVID-19 illness who required hospitalization compared to those with milder illness not requiring hospitalization (Fig. 1B). A large fraction of SARS-CoV-2-reactive CD8^+^ T cells co-expressed CD279 (PD-1), CD38 and HLA-DR, which are markers linked to T cell activation and exhaustion (*6*, *12*, *27*, *28*) (Fig. 1C, fig. S1B and table S2). Notably, we found that the proportion of SARS-CoV-2-reactive CD8^+^ T cells expressing PD-1 was not significantly different between patients with severe disease compared to mild disease (Fig. 1C). Because PD-1 is expressed by recently activated as well as exhausted T cells (*29*–*32*), it is not possible to accurately assess the exhaustion status of CD8^+^ T cells based on its surface expression levels, as proposed by other reports on global CD8^+^ T cell populations from COVID-19 patients (*12*, *28*). Recent studies in patients with COVID-19 illness have reported that circulating CD8^+^ T cells express various activation markers such as CD137, CD69, PD-1, HLA-DR and CD38, most likely activated by SARS-CoV-2 infection in vivo (*5*, *9*); these cells are also captured and contribute to an unbiased evaluation of virus-reactive T cells from patients with recent COVID-19 illness.

To study the properties of SARS-CoV-2-reactive CD8^+^ T cells in healthy non-exposed individuals (*5*, *33*, *34*), we isolated CD8^+^ T cells responding to SARS-CoV-2 peptide pools from 4 healthy subjects, who provided blood samples pre-COVID-19 pandemic (Fig. 1A and table S1). To contextualize our data and to define shared or distinguishing properties of CD8^+^ T cells reactive with other common non-CoV respiratory viruses, we stimulated PBMC from 5 healthy subjects with peptide pools specific to respiratory syncytial virus (RSV) and influenza A (FLU), and isolated responding cells (Fig. 1A). In total, we sorted and analyzed the single-cell transcriptome and T cell receptor (TCR) sequence of > 84,000 virus-reactive CD8^+^ memory T cells (stimulated in vitro) from 49 subjects (Fig. 1A, fig. S1C, D and table S3).

### Virus-reactive CD8^+^ T cells show transcriptomic heterogeneity

**Fig. 2:**
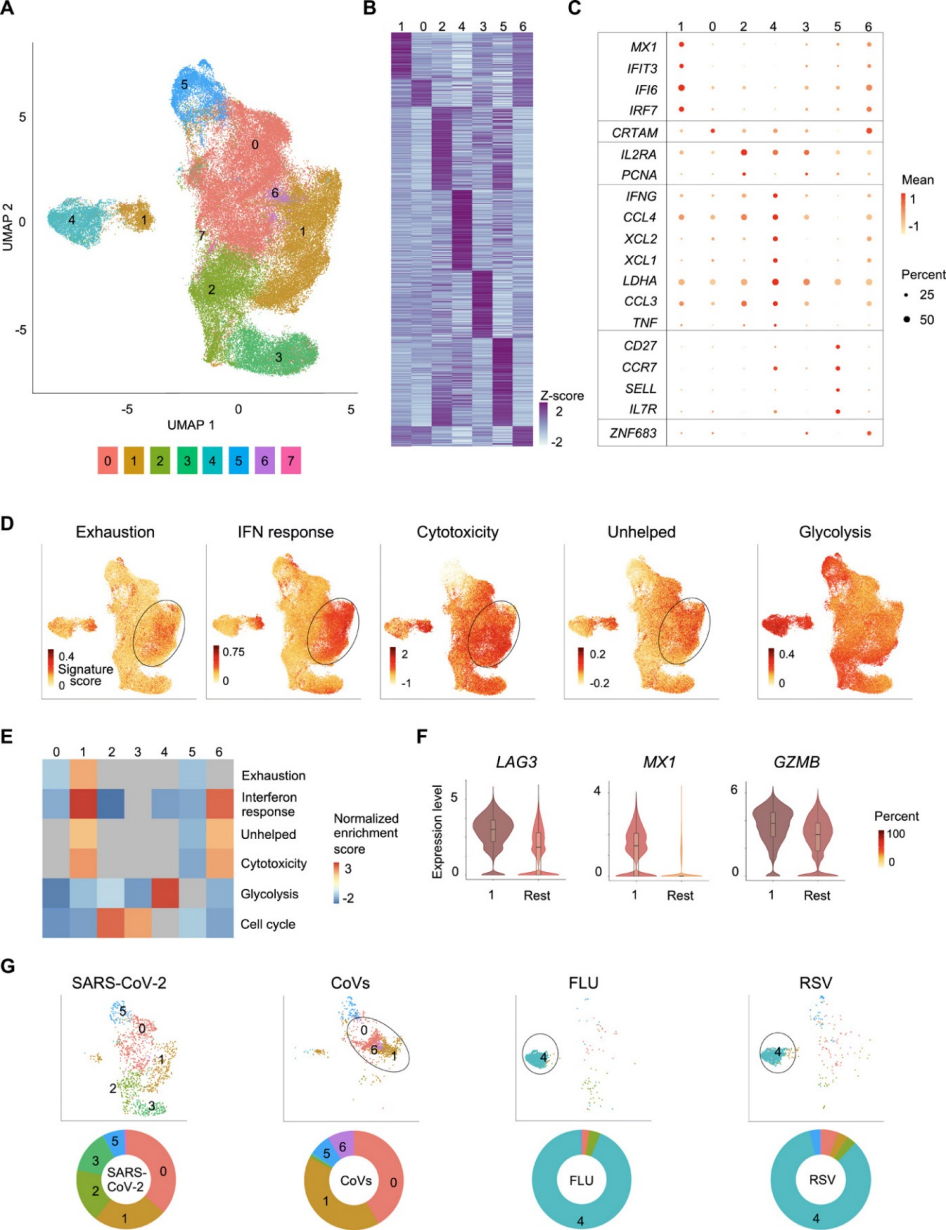
Virus-reactive CD8^+^ T cells show transcriptomic heterogeneity. (**A**) Uniform manifold approximation and projection (UMAP) analysis that displays single-cell transcriptomic landscape of sorted CD137**^+^**CD69**^+^** CD8**^+^** memory T cells following 24 hours of stimulation with virus-specific peptide pools. Seurat-based clustering of 84,140 single cells colored based on cluster type. (**B**) Heatmap showing expression of the most significantly enriched transcripts in clusters 0-6 (see table S4, Seurat marker gene analysis – comparison of a cluster of interest versus all other cells). Shown are a subset of the top 200 transcripts that have an adjusted *P* < 0.05, log_2_ fold change > 0.25, and >10% difference in the percentage of cells expressing the differentially expressed transcript between two groups compared. (**C**) Graph showing average expression (color scale) and percent of expressing cells (size scale) of selected marker transcripts in each cluster; cells in cluster 7 that comprise <1% of all cells are not shown (**B**, **C**). (**D**) UMAPs are illustrating exhaustion, interferon (IFN) response, cytotoxicity, ‘unhelped’, and glycolysis signature scores for each cell. (**E**) Gene Set Enrichment Analysis (GSEA) for the indicated gene signatures comparing each cluster with the rest of the cells. Heatmap shows summary of the normalized enrichment scores for each cluster. Gray color indicates that the signature does not reach statistical significance (*P* >0.05) in a given cluster. (**F**) Violin plots showing normalized expression level (log_2_(CPM+1)) of representative exhaustion, IFN response and cytotoxicity marker transcripts (*LAG3*, *MX1* and *GZMB*, respectively) in cluster 1 compared to an aggregation of remaining cells (Rest). Color indicates percentage of cells expressing indicated transcript. (**G**) UMAPs are depicting CD8^+^ memory T cells for individual virus-specific pool stimulation conditions (top panel). Each group of virus-reactive cells was randomly down-sampled to ensure equal representation; corresponding pie charts are displaying proportions of virus-reactive cells in individual clusters (bottom panel).

Our unbiased single-cell transcriptomic analysis of all the virus-reactive CD8^+^ T cells revealed 8 distinct clusters (Fig. 2A-C and table S4), indicating that CD8^+^ memory T cells can activate a wide range of transcriptional programs in response to different viral infections (*35*). Cluster 7 comprised less than 1% of all cells and, due to its small size, was excluded from further analysis. Recent reports from COVID-19 patients have suggested the presence of exhaustion-related markers in global CD8^+^ T cell populations based on the expression of a few coinhibitory receptors, including PD-1 (*27*, *36*–*38*). Although the increased expression of inhibitory receptors is often indicative of their ‘exhaustion’ status, recent single-cell sequencing and functional T-cell studies in human and mouse tumors reported that these inhibitory receptor-expressing T cells were highly proliferative and capable of exerting tumor control. Indeed, the present definitions of ‘T cell exhaustion’ vary from complete absence of effector functions to a state of altered functionality to limit host tissue damage (*39*). To examine, whether such ‘exhausted’ cells were present in our dataset, we first generated a consensus list of ‘exhaustion’ signature genes (n=62) from 9 studies (*31*, *32*, *40*–*46*) that reported exhaustion features in T cells analyzed from patients with infection or cancer and in mouse models of viral infection (table S5). Gene set enrichment analysis (GSEA) of individual clusters showed significant positive enrichment of exhaustion signature genes in cluster 1 cells and negative enrichment in cluster 0 cells (Fig. 2D-F and fig. S2A). Although there was no difference in expression of PD-1 in any particular cell cluster, the proportion of cells expressing several exhaustion signature genes (*e.g.*, *HAVCR2* (TIM-3), *LAG3*, *CD38*, *ISG15*, *IFI35*) was increased in cluster 1 compared to cluster 0 or other clusters (Fig. 2F, fig. S2B, C, and table S10).

In the context of murine LCMV infection persistent type I interferon signaling has been directly linked to the development of the exhaustion program in T cells (*47*–*49*). Our GSEA showed strong enrichment of genes linked to type I interferon signaling in cluster 1, which suggested that persistent type I interferon signaling may be linked to the exhaustion status of cells in cluster 1 (Fig. 2D-F and fig. S2A). In addition to interferon signaling, the exhaustion program in CD8^+^ T cells has been tightly linked to defective CD4^+^ T cell-mediated help, a multi-faceted process in which CD4^+^ T cells provide support for the differentiation and maintenance of robust CD8^+^ memory T cell responses in infection (*50*–*53*). Recent studies suggested that in the absence of CD4^+^ T cell-mediated help, ‘unhelped’ CD8^+^ T cells down-regulate the expression of genes involved in cell survival and effector functions and up-regulate the expression of genes encoding coinhibitory receptors linked to exhaustion (*52*–*54*). Because CD4^+^ T cell-mediated help is required for the generation of robust CD8^+^ memory T cells (*50*), we assessed if cells in the ‘exhausted’ cluster 1 were also displaying ‘unhelped’ features that are linked to the lack of CD4^+^ T cell ‘help’ (*50*). As expected, the ‘exhausted’ cluster 1 was also significantly enriched for transcripts linked to ‘unhelped’ CD8^+^ T cells (Fig. 2D, E and fig. S2A). Together, these findings suggested that persistent type I interferon signaling and a lack of CD4^+^ T cell ‘help’ may play a role in establishing the exhaustion program in a subset of virus-reactive CD8^+^ memory T cells. Despite displaying ‘exhausted’ and ‘unhelped’ features, intriguingly, cells in cluster 1 showed significant positive enrichment of cytotoxicity signature genes and higher expression levels of cytotoxicity-associated transcripts such as *GZMB, GZMA, GZMH*, *PRFI* and *TBX21* (Fig. 2D-F and fig. S2A, B), which suggested potential heterogeneity within this ‘exhausted’ subset. This finding is not unexpected as prior functional studies of exhausted T cells have shown that unlike cytokine production and proliferative ability, cytotoxicity features are not diminished in exhausted T cells (*40*–*43*, *55*, *56*). Furthermore, substantial heterogeneity in the nature of exhausted cells with a broad spectrum of functional capabilities has been reported (*43*, *56*–*59*).

We next assessed if the exhaustion program is associated with any impairment in the generation of effector memory cells in SARS-CoV-2 infection. Among patients with COVID-19, we found a significant negative correlation between proportion of cells in the exhausted cluster 1 and the absolute numbers of SARS-COV-2-reactive memory CD8^+^ T cells present in circulation (per million PBMCs), highlighting a functional defect in exhausted cells that potentially impacts their proliferation and persistence in vivo (fig. S2D).

Compared to direct ex vivo analysis of resting CD8^+^ T cells, the ARTE assay, which requires stimulation of TCR by MHC-peptide complex for 24 hours in vitro, has the advantage of capturing CD8^+^ T cells that respond to a wide array of SARS-CoV-2 peptides with high specificity. Although physiological stimulation of T cells provides a unique opportunity to assess molecular properties that are evident only following encounter with cognate antigen, it can also alter the transcriptional signatures of viral-reactive T cells present in vivo and thus may confound the analysis of their transcriptional signatures. Because all cells are subjected to similar stimulation conditions in the ARTE assay, the transcriptional differences that are observed between cell subsets (clusters) or patient groups are likely to be biologically relevant (*60*–*64*). Notably, we found that activation signature genes induced by TCR stimulation (*65*) were enriched to similar levels in cells from both cluster 1 (exhausted) and cluster 0 (non-exhausted), suggesting that transcriptional changes induced by in vitro stimulation are insufficient to explain the strong enrichment of exhaustion, interferon signaling and ‘unhelped’ gene signatures specifically in the ‘exhausted’ cluster 1 when compared to the ‘non-exhausted’ cluster 0 (fig. S2A).

Next, we assessed if SARS-CoV-2-reactive CD8^+^ T cells with exhausted features were also present in vivo, *i.e.*, even among cells not subjected to in vitro stimulation. Recent studies in patients with COVID-19 illness have shown that circulating CD8^+^ T cells expressing a combination of activation markers are enriched for SARS-CoV-2 reactive cells (*6*, *12*, *27*, *28*). To determine the specificity and molecular features of such T cells expressing activation markers ex vivo, we directly sorted CD38^+^HLA-DR^+^PD-1^+^ CD8^+^ memory T cells from 21 matched patients with severe COVID-19 illness and performed single-cell transcriptome and TCR sequence analysis of >20,000 cells (fig. S3B). CD8^+^ memory T cells expressing activation markers ex vivo clustered into 6 distinct subsets, and GSEA showed that a subset of cells (cluster F) was significantly enriched for ‘exhaustion’ signature genes (fig. S3C, D). This finding suggested that our strategy to isolate SARS-CoV-2-reactive CD8^+^ T cells by stimulating in vitro with SARS-CoV-2 peptides (ARTE assay) does not significantly alter the baseline ex vivo cell states, such as ‘exhaustion’ status, as this state is also observed in CD8^+^ T cells ex vivo.

Comparison of single-cell TCR sequences between CD8^+^ T cells expressing activation markers ex vivo and in vitro activated SARS-CoV-2 reactive CD8^+^ T cells from matched patients showed a large fraction of cells shared TCRs in both data sets, suggesting enrichment of SARS-CoV-2 reactive cells in ex vivo activated CD8^+^ T cells (fig. S3E). As expected, cells with shared TCR sequences showed greater clonal expansion when compared to cells not sharing TCR sequences in ex vivo and in vitro activated conditions (fig. S3F). Notably, the majority of the TCR clonotypes present in in vitro activated SARS-CoV-2 reactive CD8^+^ T cells were not captured by ex vivo activated cells (fig. S3E), which indicated that analysis of CD8^+^ T cells expressing activation markers ex vivo is not likely to fully capture the breadth of CD8^+^ T cell responses to SARS-CoV-2. Thus, while enrichment of SARS-CoV-2-reactive CD8^+^ T cells based on the co-expression of multiple activation markers expression can capture a sizeable fraction of SARS-CoV-2-reactive CD8^+^ T cells, specific stimulation in vitro with SARS-CoV-2 peptides yields an expanded spectrum of SARS-CoV-2-reactive CD8^+^ T cell responses.

CD8^+^ T cells reactive to specific viruses made strikingly different contributions to individual clusters (Fig. 2G and table S3). Clusters 0, 1, 2 and 3 were overrepresented by SARS-CoV-2-reactive CD8^+^ T cells from COVID-19 patients and are likely to reflect memory/effector cells generated in the context of a recent infection. In contrast, the vast majority (>80%) of FLU-reactive and RSV-reactive CD8^+^ T cells were present in cluster 4, where SARS-CoV-2-reactive cells were underrepresented (<2%) (Fig. 2G). Cells in cluster 4 expressed higher levels of transcripts encoding for cytokines such as IFN-γ, TNF-α, CCL3, CCL4, XCL1, and XCL2 (Fig. 2C, fig. S2E and table S4), resembling polyfunctional CD8^+^ T cells that have been linked to protective immunity toward a range of viral infections (*66*–*69*). In addition, this cluster displayed positive enrichment and the highest score for genes in the aerobic glycolysis pathway, which is linked to better effector function through multiple mechanisms that are independent of metabolism itself (reviewed in (*70*, *71*)) (Fig. 2D, E and fig. S2A).

SARS-CoV-2-reactive CD8^+^ T cells from healthy non-exposed subjects, presumed to be human CoV-reactive cells that cross-react with SARS-CoV-2 (*34*), were mainly present in clusters 0, 1, 5 and 6 (Fig. 2G and table S3). The existence of exhausted SARS-CoV-2-reactive cells (cluster 1) in healthy non-exposed subjects is in keeping with prior reports that exhausted memory cells can persist even in the absence of on-going infection or following vaccination (*50*, *52*, *53*, *72*–*75*). It is noteworthy to mention that we observe exhausted SARS-CoV-2-reactive cells in some COVID-19 patients later in the course of illness (> 3 weeks) (fig. S3G and S4A), suggesting that exhausted memory cells can be detected even when infection has been cleared. The cells in cluster 6 were highly enriched for the expression of transcripts encoding ZNF683 (fig. S3A), also known as HOBIT, a transcription factor that plays a pivotal role in the development of tissue-resident memory (T_RM_) cells (*76*). Notably, the vast majority of cells in cluster 6 were from SARS-CoV-2-reactive cells of healthy non-exposed subjects (Fig. 2G), in contrast, cells from COVID-19 patients were largely absent in this cluster. Overall, our data revealed substantial heterogeneity in the nature of CD8^+^ T cell subsets generated in response to different viral infections.

### Exhausted SARS-CoV-2-reactive CD8^+^ T cells are increased in mild COVID-19 illness

**Fig. 3:**
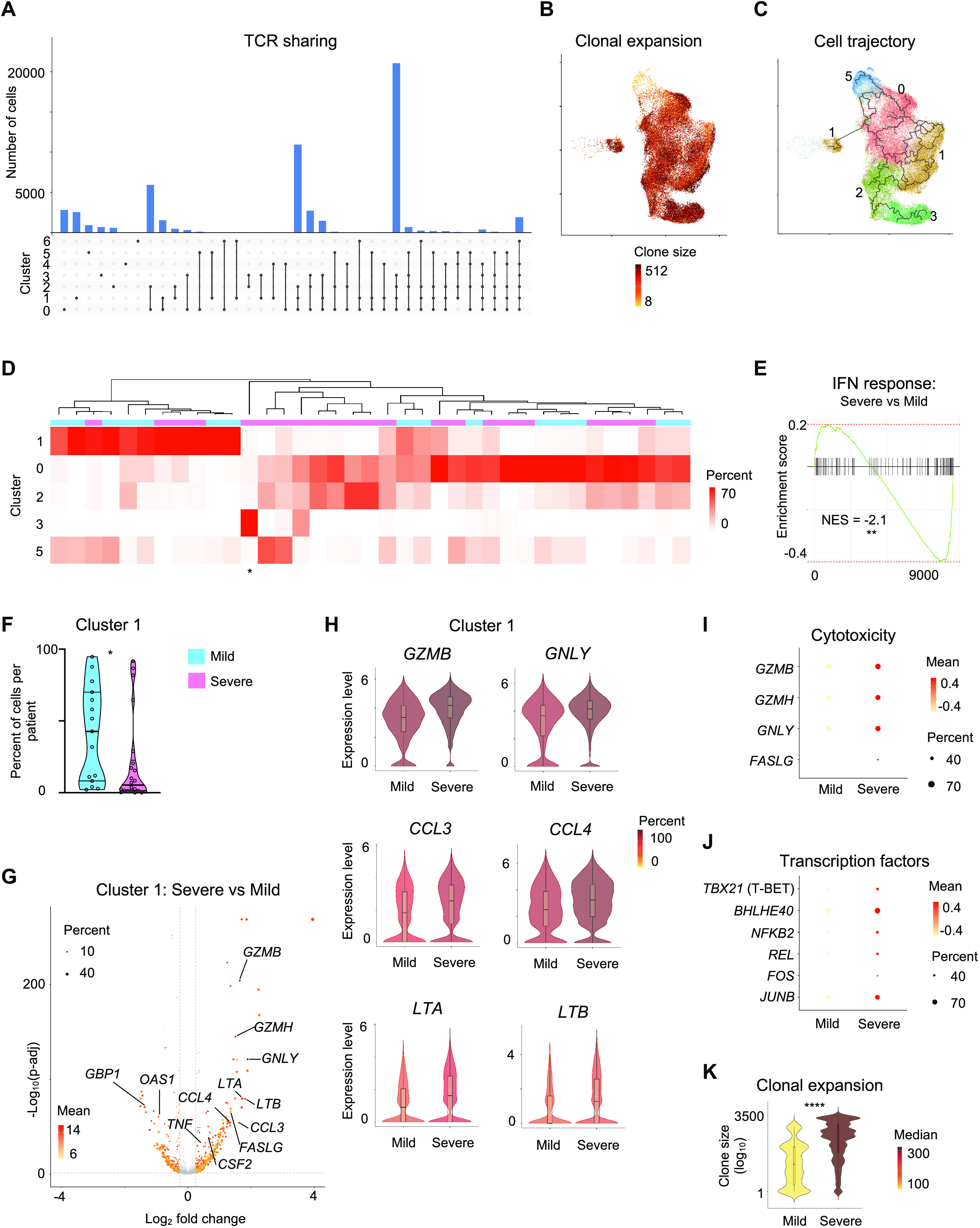
Exhausted SARS-CoV-2-reactive CD8^+^ T cells are increased in mild COVID-19 illness. (**A**) Single-cell TCR sequence analysis of SARS-CoV-2-reactive cells showing the sharing of TCRs between cells from individual clusters (rows, connected by lines). Bars (top) indicate the number of cells intersecting indicated clusters (columns). (**B**) UMAP is showing the clone size of SARS-CoV-2-reactive cells from COVID-19 patients. (**C**) Single-cell trajectory analysis showing the relationship between cells in different clusters (line). (**D**) Unsupervised clustering of all COVID-19 patients (mild and severe illness) based on the proportion of SARS-CoV-2-reactive CD8^+^ T cells present in each cluster per patient. The symbol * below represents patient 8. Clusters 4, 6, and 7 that had a very low frequency of cells in COVID-19 patients (<1% cells per cluster in total) are not shown here; full details provided in table S3. **(E)** Gene Set Enrichment Analysis (GSEA) of type I interferon response genes in all cells from COVID-19 patients with severe versus mild illness. Normalized Enrichment Score (NES) = -2.1, ** *P*<0.01. (**F**) Violin plots comparing the proportion of cells in cluster 1 from COVID-19 patients with mild and severe illness. Data are displayed as median with interquartile ranges (*n*=37, 2 subjects without hashtag data were not included for donor-specific analysis). (**G**) Volcano plot showing genes differentially expressed (adjusted *P* < 0.05, mean CPM >0, log_2_ fold change >0.25) in cluster 1 cells between COVID-19 patients with severe and mild disease. (**H**) Violin plots comparing the normalized expression level (log_2_(CPM+1)) of indicated transcripts in cluster 1 cells between COVID-19 patients with mild and severe disease. Color indicates percentage of cells expressing indicated transcript. (**I**) Plot displaying the mean expression (color scale) and percent of expressing cells (size scale) of several cytotoxicity molecules in cluster 1 cells from COVID-19 patients with severe and mild illness. (**J**) Plot displaying the mean expression (color scale) and percent of expressing cells (size scale) of several key transcription factors in cluster 1 cells from COVID-19 patients with severe and mild illness. (**K**) Violin plots showing the degree of CD8^+^ T cell-clonal expansion in cluster 1 cells between COVID-19 patients with mild and severe disease. Color indicates median size per group. * *P*<0.05, ****P<0.0001 by Mann-Whitney tests (**F**, **K**).

By linking single-cell transcriptome with T cell receptor (TCR) sequence data of the same cells, we observed extensive clonal expansion as well as clonal sharing of TCRs between the different SARS-CoV-2-reactive subsets in COVID-19 patients (Fig. 3A, B and tables S6, 7). Single-cell trajectory analysis showed that cells in cluster 0, 1 and 2 were interconnected rather than following a unidirectional path, suggesting that precursor cells with the same TCR sequence can differentiate into diverse molecular subsets (Fig. 3C). Our data supports both diversity as well as plasticity in the nature of CD8^+^ memory T cell responses to SARS-CoV-2 infection. However, the dominant memory subsets varied substantially across COVID-19 patients with cells in some subsets represented only by a few patients (Fig. 3D and table S3). For example, over 85% of SARS-CoV-2-reactive CD8^+^ T cells in patient 8 were just from cluster 3 (identified by * in Fig. 3D), indicating a lack of plasticity in CD8^+^ T cell responses in this person.

Multiple studies in patients with COVID-19 have shown impairment or defects in type I interferon signaling in patients with severe compared to mild illness (*77*–*80*). Therefore, we asked if impairment in interferon signaling pathways was observed in SARS-CoV-2 reactive CD8^+^ T cells. GSEA confirmed significant negative enrichment of type I interferon signaling genes in SARS-CoV-2 reactive CD8^+^ T cells from patients with severe illness compared to those with mild illness (Fig. 3E). Because of the positive association between persistent interferon signaling and exhaustion program in T cells (*47*–*49*), a feature we also observed in a subset of SARS-CoV-2 reactive CD8^+^ T cells (cluster 1), we assessed if the impairment in type I interferon signaling observed in severe illness also impacted the development of exhausted cells. COVID-19 patients broadly clustered into two groups based on whether the majority (>50%) of their CD8^+^ memory T cell responses to SARS-CoV-2 were either from cluster 1 or cluster 0 (Fig. 3D). Cluster 1 represented ‘exhausted’ cells with significant positive enrichment of both exhaustion and interferon signatures, whereas cluster 0 cells showed significant negative enrichment of these signatures *i.e.*, were not ‘exhausted’ (Fig. 2D, E). Our analysis suggested that a subgroup of COVID-19 patients (30%) mounted a predominantly ‘exhausted’ CD8^+^ T cell memory response to SARS-CoV-2. The magnitude of this ‘exhausted’ response showed no significant correlation to the time interval between onset of illness and sample collection (fig. S4A). Importantly, patients with milder disease had a significantly greater frequency of cells in the ‘exhausted’-interferon enriched cluster (cluster 1) when compared to those with severe disease (mean 41% versus 20%, Fig. 3F), presumably reflecting the impairment in type I interferon signaling in patients with severe disease (*77*–*80*). Additionally, patients with severe disease when compared to those with mild disease showed significant enrichment of cytotoxicity and exhaustion signature genes, and depletion of interferon signature genes in cluster 1 (fig. S4B, C), suggesting both quantitative and qualitative differences in cells in the ‘exhausted’ cluster based on disease severity. It is possible that patients with severe disease might develop a stronger ‘exhaustion’ program as a compensatory mechanism to make up for their depleted quantity of protective ‘exhausted’ cells to prevent further immunopathology while retaining their core cytotoxic functionality (fig. S4B). In support of qualitative differences, single-cell differential gene expression analysis showed that cells in the ‘exhaustion’ cluster (cluster 1) from severe COVID-19 patients expressed significantly higher levels of transcripts encoding for cytotoxicity-associated molecules (granzyme B, granzyme H, granulysin, Fas ligand (*5*, *81*)) and proinflammatory cytokines (CCL3, CCL4, CSF-2, TNF-α, LTA and LTB (*5*, *9*, *82*) (Fig. 3G-I, fig. S4D, E). Transcripts encoding for several transcription factors that support cytokine production, inflammation and persistence (T-BET, BHLHE40, NFKB2, REL, FOS, JUNB) (*83*–*88*) (Fig. 3J, fig. S4E, table S8) were also expressed at significantly higher levels in cluster 1 cells from COVID-19 patients with severe illness. TCR sequence analysis of cells in the ‘exhausted’ cluster 1 revealed greater clonal expansion in patients with severe compared to mild illness, suggesting greater proliferative capacity and/or persistence of cells from severe COVID-19 patients (Fig. 3K). Given the importance of exhaustion programs in preventing excessive host tissue damage in viral infections (*89*, *90*), we speculate that the failure to imprint an ‘exhaustion’ program that impairs T cell effector function may reflect a failure to limit exaggerated CD8^+^ T cell effector function, and thereby contribute to disease pathogenesis in some patients with severe COVID-19 illness.

### Pro-survival features are present in SARS-CoV-2-reactive CD8^+^ T cells from patients with severe COVID-19

SARS-CoV-2-reactive cells from COVID-19 patients, who did not mount a predominant ‘exhausted’ response, were present mainly in clusters 0 and 2, the ‘non-exhausted’ subsets (Fig. 2D, 3D). These ‘non-exhausted’ subsets displayed cytotoxicity signature scores comparable to other clusters (Fig. 2D). Furthermore, cluster 2 was enriched for cell cycle signature, indicative of a greater proportion of proliferating cells in this cluster (Fig. 2C, E). We found no significant difference in the proportions of cells in cluster 0 and 2 between patients with severe and mild COVID-19 illness (fig. S5A).

**Fig. 4:**
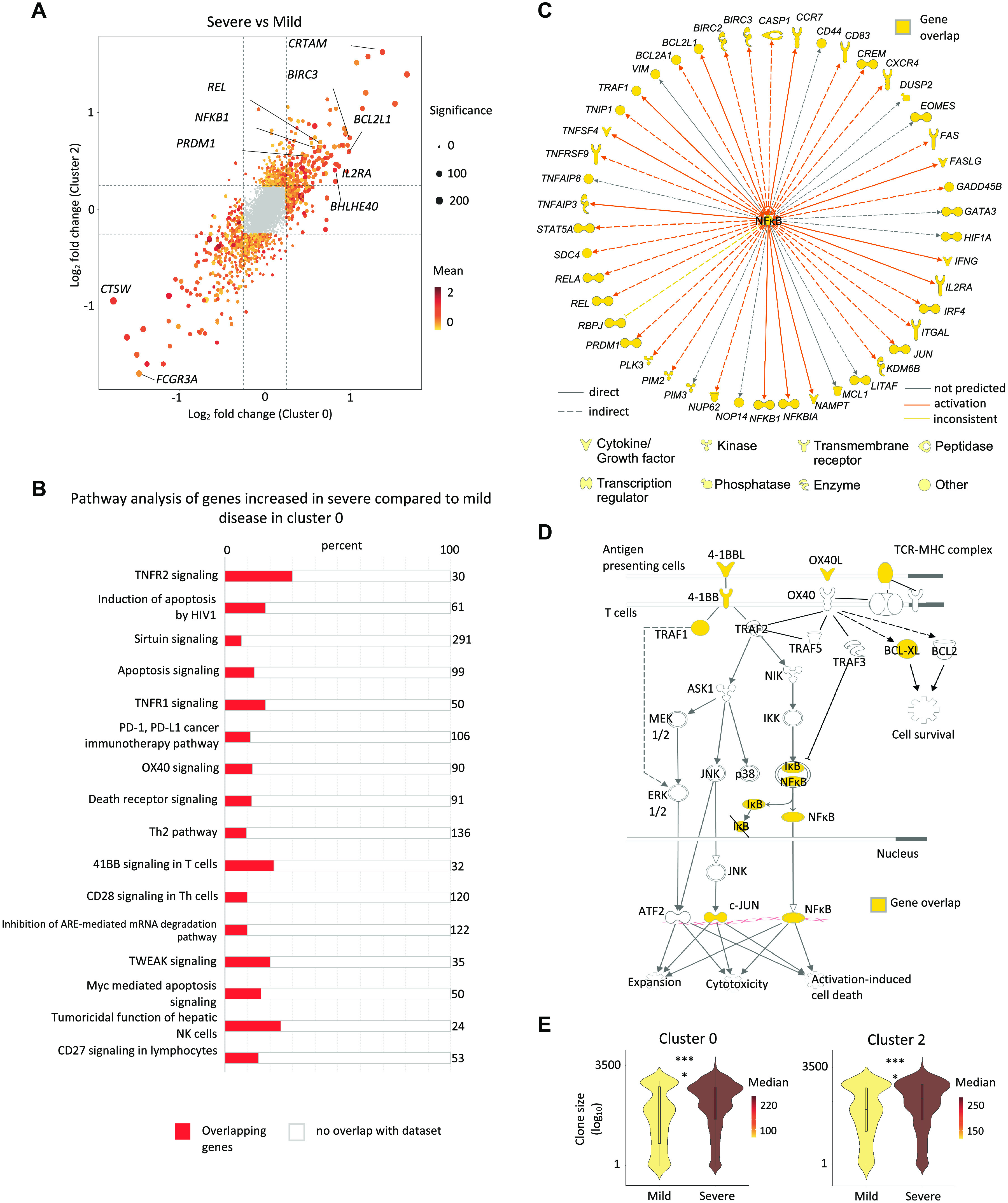
Pro-survival features in SARS-CoV-2-reactive CD8^+^ T cells from patients with severe COVID-19 illness. (**A**) Plot shows fold change values of differentially expressed genes (adjusted *P* < 0.05, mean CPM >0, log_2_ fold change >0.25) in clusters 0 (*x*-axis) and 2 (*y*-axis) when comparing COVID-19 patients with severe and mild illness. A positive value indicates that the particular gene has increased expression in patients with severe disease relative to patients with mild disease in a given cluster, while a negative value indicates the opposite. (**B**-**D**) Ingenuity Pathway Analysis (IPA) of genes with increased expression (adjusted *P* <0.05, log_2_ fold change >0.25) in cluster 0 cells between COVID-19 patients with severe versus mild illness; (**B**) Top 16 canonical pathways with significant enrichment. **(C)** Upstream regulatory network analysis of genes in NF-κB pathway. (**D**) Transcripts encoding components in the 4-1BB and OX40 signaling pathway. (**E**) Violin plots showing the degree of CD8^+^ T cell-clonal expansion in cluster 0 (left) and 2 (right) between COVID-19 patients with mild and severe disease. Color indicates median size per group. **** *P* <0.0001 by Mann-Whitney test.

However, single-cell differential gene expression analysis in cluster 0 as well as cluster 2 revealed major transcriptional differences between COVID-19 patients with mild and severe illness (Fig. 4A and table S8). Ingenuity Pathway Analysis of transcripts with increased expression in cluster 0 from COVID-19 patients with severe relative to mild illness showed significant enrichment of transcripts in multiple co-stimulation pathways (OX40, CD27, CD28, 4-1BB, CD40), the NF-κB and apoptosis signaling pathways (Fig. 4B-D, fig. S5B-D and table S9). Co-stimulation is required for the robust activation and generation of memory T cell responses (*91*) and recently, a study suggested the importance of CD4^+^ T cell-mediated ‘co-stimulatory help’ in preventing CD8^+^ T cell dysfunction (*52*). Furthermore, the activation of the NF-κB pathway has been shown to be important for T cell IL-2 production, proliferation, survival, cytokine production and effector function (*84*). IL2Rα and STAT5-encoding transcripts were also increased in severe compared to mild disease, indicating greater potential for these cells to receive the pro-survival IL-2 signals (*92*) (Fig. 4A, C). Other transcripts with increased expression encoded for transcription factors involved in cell fitness and cytokine production (BHLHE40) (*83*), effector differentiation (BLIMP1) (*93*) and prevention of exhaustion program in certain contexts (JUN) (*87*), and for CRTAM that has previously been shown to be important for generating effective cytotoxic T cells and viral clearance in mouse models (*65*, *94*) (Fig. 4A, C, D). In addition, many transcripts encoding for molecules involved in cell survival and preventing apoptosis like BIRC2, BIRC3, BCL2A1, MCL1, VIM, and BCL2-xL (*95*, *96*) were also increased in cells from patients with severe COVID-19 illness, although some molecules with pro-apoptotic function (*e.g.*, CYCS, BIM, FAS) (*97*) were also increased (Fig. 4A-D and fig. S5B). To determine the net impact of the expression of these molecules with opposing functions in the apoptosis pathway, we assessed co-expression of these genes in each cell in cluster 0. Only a small fraction of cells exclusively expressed genes with pro-apoptotic function, whereas the vast majority of cells in the cluster 0 displayed robust expression of multiple genes with pro-survival roles (fig. S5C). TCR sequence analysis indicated greater clonal expansion of cells in cluster 0 and 2 from severe COVID-19 patients (Fig. 4E). Overall, our findings suggested that SARS-CoV-2-reactive CD8^+^ T cells from patients with severe COVID-19 displayed multiple features that support the generation of robust CD8^+^ T cell memory responses with pro-survival properties. Our hypothesis based on transcriptional signatures was supported by the finding that patients with severe COVID-19 illness had a significantly greater number of SARS-CoV-2-reactive CD8^+^ memory T cells when compared to those with mild illness, and this difference was significant in patients analyzed later (> 3 weeks) in the course of illness (fig. S5D).

## DISCUSSION

Recent studies in COVID-19 patients have verified the presence of CD8^+^ T cells that are reactive to SARS-CoV-2 (*5*, *7*, *8*). However, the nature and types of CD8^+^ T cell subsets that respond to SARS-CoV-2 and whether they play an essential role in driving protective or pathogenic immune responses remain elusive. Here, we report on single-cell transcriptome and TCR sequence analyses of >87,000 in vitro activated virus-reactive CD8^+^ T cells and >20,000 CD8^+^ T cells expressing activation markers ex vivo, from a total of 39 COVID-19 patients and 10 healthy, pre-pandemic donors. To compare the molecular properties of antigen-specific SARS-CoV-2-reactive CD8^+^ T cells to other common respiratory virus-reactive CD8^+^ T cells, we also isolated virus-reactive CD8^+^ memory T cells from healthy control subjects and analyzed their single-cell transcriptomes.

Across all the virus-reactive CD8^+^ T cells studied, we delineated eight distinct clusters with distinct transcriptomic features. TCR sharing between clusters identified a high degree of plasticity among virus-reactive CD8^+^ T cells. We find that in vitro peptide stimulation does not significantly alter the baseline ex vivo state of the viral-reactive CD8^+^ memory T cells and these cells can acquire a wide range of transcriptional programs following different viral infections. For example, in healthy subjects, CD8^+^ T cells with polyfunctional features, linked to protective anti-viral immunity (*66*–*69*), are abundant among CD8^+^ memory T cells reactive to FLU and RSV. In contrast, these cells were mostly absent in SARS-CoV-2 responsive cells from both COVID-19 patients and healthy non-exposed subjects. Notably, cells in this polyfunctional cluster were also significantly enriched for genes related to aerobic glycolysis, which is considered to enhance effector functions of CD8^+^ memory T cells (*70*, *71*). The absence of such polyfunctional memory CD8^+^ T cells in SARS-CoV-2 infection may be due to the short interval between symptom onset and blood collection for analysis (median 20 days), a time period when effector responses as opposed to long-term memory responses are likely to be captured. Long-term follow up studies will be required to clarify if SARS-CoV-2 infection generates such polyfunctional long-term memory T cells.

A large fraction of SARS-CoV-2 reactive cells (43% and 37%) from healthy non-exposed subjects (pre-pandemic), presumed to be human CoV-reactive cells that cross-react with SARS-CoV-2 peptide pools (*5*, *34*), were present in clusters 1 and 0, respectively. These clusters also had similar representation of SARS-CoV-2 reactive cells from patients with COVID-19 illness. Cells in cluster 1 showed significant positive enrichment for type I interferon signaling, CD4 T-‘unhelped’ and ‘exhaustion’ signatures, reminiscent of the ‘exhausted’ CD8^+^ T cells reported in murine LCMV infection models (*98*). Cluster 0, in contrast, was non-exhausted and showed significant negative enrichment of exhaustion and interferon signatures. SARS-CoV-2 reactive cells from COVID-19 patients also contributed the large majority of cells in cluster 2, which was characterized by enriched expression of cell cycle-related genes. Like cells in cluster 0, cluster 2 cells also showed negative enrichment of interferon genes and relatively lower exhaustion signature scores. Thus, we find that the nature of CD8^+^ T cells reactive to *Coronaviridae* differed substantially from those responding to FLU or RSV.

Intriguingly, COVID-19 patients broadly segregated into two groups, according to whether the majority of their virus-reactive CD8^+^ memory T cells were in the ‘non-exhausted’ cluster 0 or the ‘exhausted’ cluster 1. Patients with mild COVID-19 illness had a greater proportion of SARS-CoV-2 reactive cells in the ‘exhausted’ cluster 1 with the ability to maintain their ‘exhausted’ state, even after viral infection resolution. Besides, cells in the ‘exhausted’ subset (cluster 1) from patients with mild COVID-19 illness expressed significantly higher levels of type I interferon response genes (*77*–*80*), lesser levels of transcripts encoding for cytotoxicity molecules, Fas ligand and proinflammatory cytokines (CCL3, CCL4, CSF-2, TNF, LTA and LTB) (*5*, *9*, *81*, *82*), and were significantly less clonally expanded. This raises the possibility that the magnitude and quality of the ‘exhausted’ CD8^+^ T cell response may be clinically important for limiting excessive tissue damage by SARS-CoV-2-reactive CD8^+^ T cells in COVID-19 illness.

Qualitative differences in the ‘non-exhausted’ clusters 0 and 2 further emerged between patients with mild and severe COVID-19 illness. Transcripts increased in cluster 0 cells from severe relative to mild illness were significantly enriched in multiple co-stimulation pathways (OX40, CD27, CD28, 4-1BB, CD40) that are linked to CD4^+^ T cell-mediated ‘helped’ features (*52*), NF-κB and cell survival pathways thought to be important for IL-2 production, proliferation, and survival. This finding suggested patients with severe disease mount a more effective CD8^+^ memory T cell response to SARS-CoV-2 infection that could potentially lead to durable protection against re-exposure. Indeed, recent studies (*8*, *12*, *14*, *17*) highlighted that convalescent COVID-19 patients with history of severe disease mounted more robust CD8^+^ memory T cell response to SARS-CoV-2 infection that could potentially lead to durable protection against re-exposure. Overall, our findings indicate that SARS-CoV-2-reactive CD8^+^ T cells from patients with severe COVID-19 displayed multiple features that support the generation of robust CD8^+^ T cell memory responses with pro-survival properties and a lack of “restrained function” via ‘exhaustion’ features. Whether these cells play a role in disease pathogenesis or provide long-term immunity is not clear, and further longitudinal analysis and function studies in relevant model organisms are required to clarify this.

## MATERIALS AND METHODS

### Patient recruitment, ethics approval and sample processing

The Ethics Committee of La Jolla Institute (USA) and the Berkshire Research Ethics Committee (UK) 20/SC/0155 provided ethical approval for this study with written consent from all participants. 22 hospitalized patients with reverse transcriptase polymerase chain reaction (RT-PCR) assay confirmed SARS-CoV-2 infection were recruited between April-May 2020. A cohort of 17 non-hospitalized participants were also recruited with RT-PCR assay or serological evidence of SARS-CoV-2 infection. Up to 80 ml of blood was obtained from all subjects for this research. Clinical metadata linked to hospitalized patients such as age, gender, comorbidities, level of clinical care required, radiological findings and laboratory results are provided in table S1. The COVID-19 cohort consisted of 30 (77%) White British/White Other, 4 (10%) Indian, 2 (5%) Black British, 2 Arab (5%) and 1 Chinese (3%) participants. Of the 39 COVID-19 subjects, 22 (56%) had moderate/severe disease requiring hospitalization and 17 (44%) had mild disease, not requiring hospitalization. The median age of the hospitalized patients was 60 (33-82) and 77% were male. The median age of the non-hospitalized participants was 39 (22-50) and 47% were male. To study SARS-CoV-2, FLU and RSV-reactive CD8^+^ T cells from healthy subjects, we utilized de-identified buffy coat samples from healthy adult subjects who donated blood at the San Diego Blood Bank before 2019, prior to the COVID-19 pandemic. Peripheral blood mononuclear cells (PBMCs) were isolated from blood by density centrifugation over Lymphoprep (Axis-Shield PoC AS, Oslo, Norway) and cryopreserved in 50% human serum, 40% complete RMPI 1640 medium and 10% DMSO.

### Peptide pools

Two peptide pools (Miltenyi Biotec) consist of lyophilized peptides (15-mer sequences) with 11 amino acids overlap. For the S protein the peptides cover Spike glycoprotein of SARS-CoV-2 domains aa 304–338, 421–475, 492–519, 683–707, 741–770, 785–802, and 885–1273 (PepTivator SARS-CoV-2 Prot_S). For the M protein the peptide pools cover the entire 221 sequence membrane glycoprotein (“M”) of SARS-CoV-2 (GenBank MN908947.3, Protein QHD43419.1) (PepTivator SARS-CoV-2 Prot_M). Our analysis (fig. S1E) showed that both M-reactive and S-reactive CD8^+^ T cells were evenly distributed across all of the clusters of viral-reactive T cells, which indicated relatively similar transcriptional patterns of S- and M-reactive cells. For capturing FLU- and RSV-reactive CD8^+^ T cells, PepTivator Influenza A (H1N1) and RSV strain B1 peptide pools that covered the entire sequence of Hemagglutinin (HA) and Nucleoprotein (N) of each virus respectively, were obtained from Miltenyi Biotec.

### Antigen-reactive T cell enrichment (ARTE) assay

Virus-reactive CD8^+^ memory T cells were isolated using the protocol from Bacher *et al*. (*24*) with minor modifications. Thawed PBMC were sorted with FACSAria Fusion Cell Sorter (Becton Dickinson) to retrieve ex vivo pre-activated CD8^+^ T cells or plated overnight (5% CO_2_, 37°C) in 1 ml (concentration of 5 × 10^6^ cells/ml) of TexMACS medium (Miltenyi Biotec) on 24-well culture plates. Each of the SARS-CoV-2-specific peptide pools (1 μg/ml) were added separately to the PBMC culture for 24 hours. For subsequent magnetic-based enrichment of CD137^+^ cells, cells were sequentially stained with human serum IgG (Sigma Aldrich) for FcR block, cell viability dye (eFluor780/APC.Cy7, eBioscience), fluorescence-conjugated antibodies, Cell-hashtag TotalSeq-C antibody (0.5 μg/condition, clone: LNH-94;2M2, Biolegend), and a biotin-conjugated CD137 antibody (clone REA765; Miltenyi Biotec) followed by anti-biotin microbeads (Miltenyi Biotec). The following fluorescence-conjugated antibodies were used: anti-human CD3 (UCHT1, Biolegend), CD4 (OKT4, Biolegend), CD8B (SIDI8BEE, eBioscience), CD137 (4B4-1, Biolegend), CD69 (FN50, Biolegend), CCR7 (3D12, BD Biosciences), CD45RA (HI100, Biolegend), CD38 (HB-7, Biolegend), HLA-DR (G46-6, BD Biosciences), PD-1 (EH12.1, Biolegend). Antibody-tagged cells were added to MS columns (Miltenyi Biotec) to positively select CD137^+^ cells. After elution, FACSAria Fusion Cell Sorter (Becton Dickinson) was utilized to sort memory CD8^+^ memory T cells expressing CD137 and CD69. Fig. S1A shows the gating strategy used for sorting. FlowJo software (v10.6.0) was employed for all Flow Cytometry Data analysis. In parallel, virus-reactive CD4^+^ memory T cells were isolated from the same cultures and analysis of their single-cell transcriptomes reported elsewhere (*99*).

### Cell isolation and single-cell RNA-seq assay (10X Genomics platform)

To facilitate the integration of single-cell RNA-seq and TCR-seq profiling from the sorted CD8^+^ T cells, 10x Genomics 5′TAG v1.0 chemistry was utilized. A maximum of 60,000 virus-responsive memory CD8^+^ T cells from up to 8 donors were sorted into a low retention 1.5mL collection tube, containing 500 μL of a solution of PBS:FBS (1:1) supplemented with RNase inhibitor (1:100). After sorting, ice-cold PBS was added to make up to a volume of 1400 μl. Cells were then spun down (5 min, 600 g, 4°C) and the supernatant was carefully aspirated, leaving 5 to 10 μl. The cell pellet was gently resuspended in 25 μl of resuspension buffer (0.22 μm filtered ice-cold PBS supplemented with ultra-pure bovine serum albumin; 0.04%, Sigma-Aldrich). Following that, 33 μl of the cell suspension was transferred to a PCR-tube and single-cell libraries prepared as per the manufacturer’s instructions (10x Genomics).

### Single-cell transcriptome analysis

Using 10x Genomics’ Cell Ranger software (v3.1.0) and the GRCh37 reference (v3.0.0) genome, reads from single-cell RNA-seq were aligned and collapsed into Unique Molecular Identifiers (UMI) counts. The Feature Barcoding Analysis pipeline from Cell Ranger was used to generate hashtag UMI counts for each TotalSeq-C antibody-capture library. UMI counts of cell barcodes were first obtained from the raw data output, and barcodes with less than 100 UMI for the most abundant hashtag were filtered out. Donor identities were assigned using Seurat’s (v3.1.5) *MULTIseqDemux* (autoThresh = TRUE and maxiter = 10) with the UMI counts. Cell barcodes were classified into three categories: donor ID (singlet), Doublet, Negative enrichment. Singlet cells were then stringently re-classified as doublet if the ratio of UMI counts between the top 2 barcodes was less than 3. All cells that were not classified as doublets or negative were used for downstream analyses. Cells from two COVID-19 patients with mild disease (patient 28 and 48) were not identifiable in the downstream analyses due to the lack of cell hashtags.

Single-cell RNA-Seq libraries (N = 4 and N = 15 for the ex vivo (0-hours) and in vitro activated (24-hours) cells, respectively) were aggregated using Cell Ranger’s *aggr* function (v3.1.0). Analysis of the combined data was carried out in the R statistical environment using the package Seurat (v3.1.5). To filter out doublets and to eliminate cells with low quality transcriptomes, cells were excluded if they were expressing < 800 or > 4400 unique genes, had < 1500 or > 20,000 total UMI content, and > 10% of mitochondrial UMIs. The summary statistics for each single-cell transcriptome library are given in table S3 and show good quality data with no major differences in quality control metrics between batches (fig. S1C). Only transcripts expressed in at least 0.1% of the cells were included for further analysis. Using default settings in Seurat software, the filtered transcriptome data was then normalized (by a factor of 10,000) and log-transformed per cell. The top variable genes with a mean expression greater than 0.01 counts per million (CPM) and explaining 25% and 16% of the total variance (for the 24- and 0-hours datasets, respectively) were selected using the Variance Stabilizing Transformation method (*100*). The transcriptomic data was then further scaled by regressing the number of UMIs detected and the percentage of mitochondrial counts per cell. This process was applied independently for the 0-hours and 24-hours datasets. Principal component analysis was performed using the top variable genes and based on the standard deviation of the principal components portrayed as an “elbow plot”, the first 16 principal components (PCs) were selected for the 0-hours dataset and the first 25 PCs were selected for the 24-hours dataset for downstream analyses. Cells were clustered using the *FindNeighbors* and *FindClusters* functions in Seurat with a resolution of 0.2 for either dataset. The robustness of clustering was verified by other clustering methods and by modifying the number of PCs and variable genes utilized for clustering. Analysis of clustering patterns of SARS-CoV-2-reactive CD8^+^ T cells across multiple batches revealed no evidence of strong batch effects (fig. S1D). Plots to visualize normalized UMI data were created using the Seurat package and custom R scripts.

### Single-cell differential gene expression analysis

MAST package in R (v1.8.2) (*101*) was used to perform pair-wise single-cell differential gene expression analysis after converting UMI data to log_2_(CPM+1). For genes to be considered differentially expressed, the following thresholds were used: Benjamini-Hochberg–adjusted *P*-value < 0.05 and a log_2_ fold change greater than 0.25. Cluster markers (transcripts enriched in a given cluster) were determined using the function *FindAllMarkers* from Seurat.

### Gene Set Enrichment Analysis and Signature Module Scores

Signature lists were extracted from published data sets and databases. Gene names from murine datasets were converted to human gene names using the biomaRt R package. Gene lists were then filtered to exclude genes that were expressed (CPM > 0) in < 2% of the cells. Exhaustion consensus signature list was derived by considering genes that were present in > 3 exhaustion signature datasets (*31*, *32*, *40*–*46*). Genes that were present in cytotoxicity signatures (*40*, *102*) or viral activation signatures (*65*) were excluded from the consensus list (table S5). The R package *fgsea* was used to calculate the GSEA scores with the signal-to-noise ratio as a metric. Default parameters other than minSize = 3 and maxSize = 500 were used. Enrichment scores for each gene signature are presented as enrichment plots. A gene signature set list is considered to be significantly enriched if adjusted *P*-value is < 0.05.

Signature scores were estimated with the Seurat’s *AddModuleScore* function, using default settings. Briefly, the signature score is defined for each cell by the mean of the gene list of interest minus the mean expression of aggregate control gene lists. Control gene lists were sampled (size equal to the signature list) from bins defined based on the level of expression of the genes in the signature list. Signature gene lists used for analysis are provided in table S5.

### Single-cell trajectory analysis

Monocle 3 (v0.2.1) (*103*) was used to calculate the “branched” trajectory, settings included the number of UMI and percentage of mitochondrial UMI as the model formula, and taking the highly variable genes from Seurat for consistency. After using the PCA output from Seurat and allocating a single partition for all cells, the cell-trajectory was outlined on the UMAP generated from Seurat as well. The ‘root’ was selected using the *get_earliest_principal_node* function given in the package’s tutorial.

### T cell receptor (TCR) sequence analysis

Single-cell libraries enriched for V(D)J TCR sequences were processed to get clonotype information for each independent sample with the *vdj* pipeline from Cell Ranger (v3.1.0 and human annotations reference GRCh38, v3.1.0, as recommended). Joint analysis of single-cell transcriptomes and TCR repertoires was performed by aggregating independent libraries through custom scripts. For this purpose, cell barcodes were matched between corresponding libraries from each type. Then, every unique clonotype, a set of productive Complementarity-Determining Region 3 (CDR3) sequences as defined by 10x Genomics, was identified across all library annotations files. Finally, clone statistics, mainly clonotypes’ frequencies and proportions, were recalculated for the whole aggregation so that previously-identified good quality cells were annotated with a specific clonotype ID and such clone statistics. Clone size was calculated as the number of cells expressing a given clonotype ID, and a clonotype was called as clonally expanded if this value was greater than 1 (clone size ≥ 2). These steps were followed either 1) independently for the 0- and 24-hours datasets (describing ex vivo and in vitro activated cells, respectively) or 2) for the whole set of cells in order to assess clonal sharing between the two conditions (tables S6 and S7). Clone size was depicted on UMAP (per cell) or in violin plots (per group, where color indicated clone size median of each group) using custom scripts and clonotype sharing was presented using the UpSetR package (*104*). For the comparison between ex vivo and in vitro activated cells, only SARS-CoV-2-reactive CD8^+^ cells specifically isolated from matched patients between datasets were considered for the 24-hours data.

### Ingenuity Pathway Analysis (IPA)

IPA was performed using default setting (v01-16) on transcripts that were significantly increased in expression in cluster 0 cells from patients with severe COVID-19 illness compared to mild illness. The canonical pathway analysis was performed to elucidate the enriched pathways in this data set and to visualize and highlight the gene overlap between the given data set with a particular enriched pathway. The upstream regulator network analysis was used to identify and visualize the interactions between differentially expressed downstream target genes on a given dataset with a particular upstream regulator.

### Statistical Analysis

GraphPad Prism 8.4.3 software was utilized for relevant data statistical analysis. Detailed information regarding statistical analysis, including test types and number of batches or samples is provided in the figure legends. P values are specified in the text or the figure legends. The data normality tests were performed and for data that fell within Gaussian distribution, appropriate parametric statistical tests were performed and for those that did not conform to the equal variance-Gaussian distribution, appropriate non-parametric statistical tests were used.

## Supplementary Materials

immunology.sciencemag.org/cgi/content/full/6/55/eabe4782/DC1 Fig. S1. CD8^+^ T cell responses in COVID-19 illness. Fig. S2. Virus-reactive CD8^+^ T cells show transcriptomic heterogeneity. Fig. S3. Analysis of CD8^+^ memory T cells expressing activation markers *ex vivo* from COVID-19 patients. Fig. S4. Exhausted SARS-CoV-2-reactive CD8^+^ cells are increased in mild COVID-19 illness. Fig. S5. Pro-survival features in SARS-CoV-2-reactive CD8^+^ T cells from patients with severe COVID-19. Table S1. Human subject details (Excel spreadsheet) Table S2. Summary of all FACS data (Excel spreadsheet) Table S3. Single-cell sequencing quality controls and subject-specific cell numbers (Excel spreadsheet) Table S4. Single-cell cluster enriched transcripts (Excel spreadsheet) Table S5. Gene lists utilized for Gene Set Enrichment Analysis and Signature Module Scores (Excel spreadsheet) Table S6. Subject-specific TCR clonotype data (Excel spreadsheet) Table S7. Cluster-specific TCR clonotype data (Excel spreadsheet) Table S8. Single-cell differential gene expression analysis (Excel spreadsheet) Table S9. Ingenuity Pathway Analysis (Excel spreadsheet) Table S10. Summary statistics for exhaustion-related genes across clusters (Excel spreadsheet) Table S11. Raw data file (Excel spreadsheet)
